# Amyand’s hernia repair in an asymptomatic patient—a case report and a review of the literature

**DOI:** 10.1093/jscr/rjae333

**Published:** 2024-05-18

**Authors:** Nikolaos V Smyrlis, Stylianos Vladimiros P Mouratidis, Panagiotis I Makedos, Konstantinos S Georgakis

**Affiliations:** Department of General Surgery, General Hospital of Chalkidiki, 63100 Polygyros, Greece; Department of General Surgery, General Hospital of Chalkidiki, 63100 Polygyros, Greece; Department of General Surgery, General Hospital of Chalkidiki, 63100 Polygyros, Greece; Department of General Surgery, General Hospital of Chalkidiki, 63100 Polygyros, Greece

**Keywords:** case report, Amyand’s hernia, mesh

## Abstract

Amyand’s hernia is a rare condition, in which the appendix is found in the inguinal hernia sac. Many patients remain asymptomatic and this pathological condition is most commonly diagnosed intraoperatively. We report a case of a 66-year-old male who was admitted to the hospital for an inguinal hernia repair and the presence of the appendix in the inguinal hernia sac was discovered intraoperatively. We performed a reduction of the appendix in the abdominal cavity and a hernia repair with a mesh and a plug. There are few studies in literature about the appropriate method of treatment and, because of that, the therapeutic approach remains controversial. Therefore, we also conducted a review of the literature in order to obtain some conclusions regarding the management of Amyand’s hernia.

## Introduction

Amyand’s hernia is defined widely as the presence of the appendix in the inguinal hernia sac, with or without inflammatory changes [1]. It is a rare condition, with an incidence below 1%. Coexistance of acute appendicitis with Amyand’s hernia is extremely rarer, approximately 0.1% of all cases of appendicitis [2]. Consequently, the majority of patients remain asymptomatic and the appendix being discovered without inflammatory changes intraoperatively during inguinal hernia repair. The intraoperative diagnosis is caused also by the fact, that it is not customary to perform a preoperative imaging investigation [3]. Several classifications have been proposed for Amyand’s hernia. The most common is that of Losanoff and Basson, as well as a modification of that, known as Rikki’s classification [4].

We present the case of a patient who was admitted to the hospital for a scheduled operation for inguinal hernia repair and an Amyand’s hernia was discovered intraoperatively.

## Case report

A 66-year-old male was admitted to our hospital for a scheduled operation due to a right inguinal hernia. One month prior to the operation, he presented in our department and complained about a swelling on the right inguinal region. He referred that it was initially smaller and gradually increased in size. The patient asked for medical advice due to local discomfort on this region for the past 5 months. The patient referred no other symptoms.

No past medical history, prescriptions or past operations were mentioned. The patient was a long-term heavy smoker.

On physical examination, an indirect incomplete inguinal hernia was discovered.

The patient was admitted to our hospital 1 month later, on the scheduled date. Before admission, he underwent a preoperative evaluation. Laboratories findings were normal and the preoperative chest X-ray revealed no pathological findings. No further imaging investigations were performed.

**Figure 1:**
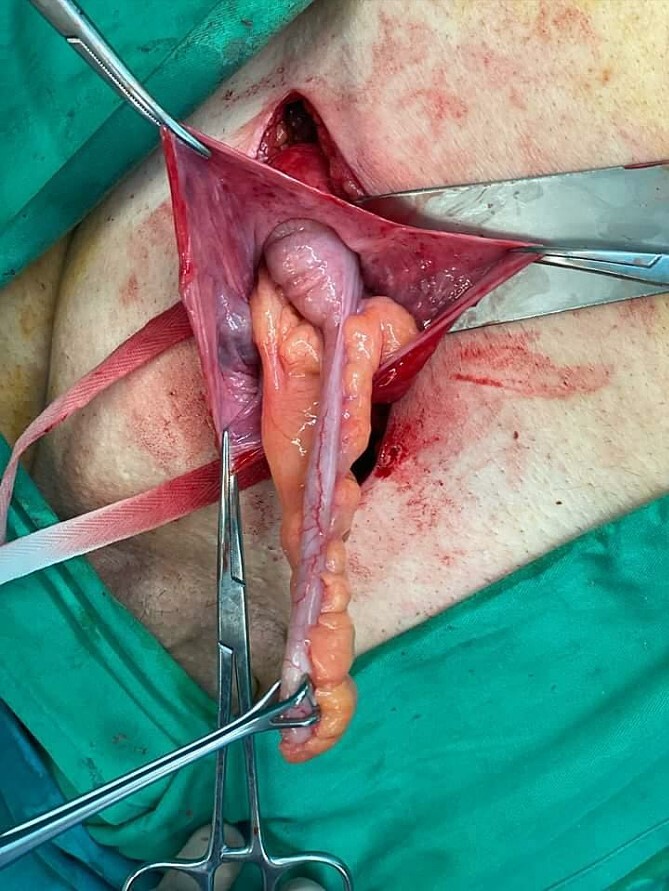
Amyand’s hernia—open approach. The hernia sac was opened and it was found to contain the appendix.

We planned to perform an inguinal hernia repair with a mesh and a plug. Under spinal anesthesia, an oblique incision was made, parallel to the inguinal ligament. The soft tissue was dissected and the dissection was continued through Camper’s fascia, as well as the more fibrous Scarpa’s fascia. After the identification of the aponeurosis of the external oblique muscle, the aponeurosis was opened and the flaps (the medial and the lateral) were elevated. The spermatic cord was explored for evidence of an indirect hernia. After the identification of the hernia, the hernia sac was separated from the cord structures down to the internal inguinal ring and was then detached from the surrounding structures. As usually, the sac was opened in order to assure that there is not a sliding component towards the hernia. The hernia sac was found to contain the appendix (Fig. 1).

The appendix was normal, without inflammatory changes. Consequently, the intraoperative diagnosis was an Amyand’s hernia Type I, according to the most commonly used classifications. Due to the lack of inflammatory changes, we preferred to perform a reduction of the appendix in the abdominal cavity and not an appendectomy. The sac was ligated and excised. After that, we performed an inguinal hernia repair with a mesh and a plug and the spermatic cord was relocated to its normal anatomic position, above the re-created floor of the inguinal canal. The external oblique aponeurosis as well as the subcutaneous tissue were closed with an absorbable suture. Finally, the skin was closed with surgical-clips.

The patient was discharged on the third-postoperative day after a normal postoperative period. He returned for a follow-up, 2 weeks after the operation, in order to remove the surgical clips. At the time of the follow-up, the patient presented without any postoperative complications.

## Discussion

Amyand’s hernia got its name thanks to Claudius Amyand, a surgeon who performed the first appendectomy on an 11-year-old patient in 1735, who presented with symptoms of acute appendicitis and a right inguinal hernia [5]. The treatment option is usually determined by the clinical presentation and the presence or not of acute appendicitis intraoperatively [6]. The appropriate therapeutic approach is still controversial.

We conducted a search through PubMed database for articles that referred to treatment options of Amyand’s hernia. We used the key words ‘Amyand’s hernia’ AND ‘mesh’ and searched for articles that were published between 2013 and 2023. The articles were also published in English language. We searched for Randomized Control Trials, Systematic Reviews and Metanalyses. It was intrigued that, during the last decade, we found only three [3] published Systematic Reviews after the search in this database.

According to Papaconstantinou et al., the majority of patients underwent appendectomy, with a rate of 58% in elective cases and 95.6% in emergent cases. A hernia repair with a mesh was preferred in elective cases (82.7%), while a herniorrhaphy was performed in the majority of patients with findings of acute appendicitis (57.4%) [7]. In the study of Manatakis et al., the majority of patients underwent an appendectomy (65% in elective and 90% in acute cases). Although a hernia repair with a mesh was performed in the majority of elective cases (62%), the use of this technique was extremely lower in acute cases (19%) [8]. Finally, according to Bratu et al., in the studied cases, appendectomy was performed in 52.73% of them. In cases with findings of appendicitis, the treatment of choice was appendectomy, while in cases in which the appendix was presented without inflammatory changes, there was no consensus between the reduction of appendix in the peritoneal cavity and the appendectomy. Also, it was interesting that a mesh repair was performed in all patients, aside from the presence or not of an inflamed appendix [9].

To sum up, the appropriate method of treatment remains controversial and more studies are required, due to the lack of them in the existing literature. This further research will assist in the establishment of certain guidelines about the most appropriate operative management for this rare condition.

## Data Availability

Data sharing is not applicable to this article as no new data were created or analyzed in this study.
